# Optimal system and dynamics of optical soliton solutions for the Schamel KdV equation

**DOI:** 10.1038/s41598-023-42477-4

**Published:** 2023-09-16

**Authors:** A. Hussain, Younes Chahlaoui, M. Usman, F. D. Zaman, Choonkil Park

**Affiliations:** 1 Abdus Salam School of Mathematical Sciences, Government College University, 68-B New Muslim Town, Lahore, 54600 Pakistan; 2 Mathematics Department, College of Science, King Khalid University, Abha, 62529 Saudi Arabia; 3 College of Electrical and Mechanical Engineering (CEME), National University of Sciences and Technology (NUST), H-12, Islamabad, 44000 Pakistan; 4 Research Institute for Natural Sciences, Department of Mathematics, Hanyang University, Seoul, 04763 South Korea

**Keywords:** Mathematics and computing, Physics

## Abstract

In this research, we investigate the integrability properties of the Schamel–Korteweg–de Vries (S-KdV) equation, which is important for understanding the effect of electron trapping in the nonlinear interaction of ion-acoustic waves. Using the optimal system, we come over reduced ordinary differential equations (ODEs). To deal with reduced ODEs for this problem, Lie symmetry analysis is combined with the modified auxiliary equation (MAE) procedure and the generalized Jacobi elliptic function expansion (JEF) method. The analytical solutions reported here are novel and have a wide range of applications in mathematical physics.

## Introduction

Many dynamical systems are commonly described using nonlinear evolution equations as models in a variety of scientific disciplines, notably fluid mechanics, plasma physics, solid-state physics, nonlinear optics, chemical kinematics, astrophysics, optical fiber, geochemistry, and chemical chemistry. To better understand the physical phenomena besides further applications in practical life, it is important to seek as many exact solutions as we can. Numerous important techniques, like the variational iteration method^1^, the sine-cosine technique^2^, the homotopy perturbation technique^3^, the first integral method^4^, the Bäcklund transformations^5^, the Jacobi elliptic function expansion procedure^6^, the $(G'/G)$-expansion method^7^, the exponential function technique^8^, the Weierstrass elliptic function method^9^, the tanh function technique and its extensions^10^, the simplest equation technique^11^ and the Lie group analysis method^12–15^ have been developed during the past few decades to provide exact solutions as well as understand their features. In this study, the S-KdV equation is taken into account^16^

1 $$\begin{aligned} U_{t}+U_{x}(\alpha _1 \sqrt{U}+\alpha _2 U)+\alpha _3 U_{xxx}=0. \end{aligned}$$

where $\alpha _1,~\alpha _2$ and $\alpha _3$ are constants. Equation (1) is relevant to the study of ion-acoustic solitons in plasma physics when electron entrapment occurs. It also controls the electrostatic potential for a specific electron distribution in velocity space. Tagare and Chakraborti demonstrated in^17^ using the direct integral approach that Eq. (1) has a single wave solution. Lee and Sakthivel provided some precise traveling wave solutions for Eq. (1) in^18^ using the exp-function approach. A generalized KdV equation is an instance of Eq. (1) that has been examined in several situations arising in mathematical physics. Equation (1) turns into the Schamel equation for $\alpha _2=0$,

2 $$\begin{aligned} U_{t}+U_{x}(\alpha _1 \sqrt{U})+\alpha _3 U_{xxx}=0. \end{aligned}$$

Whereas Eq. (1) becomes a well-known KdV equation when $\alpha _1=0$

3 $$\begin{aligned} U_{t}+\alpha _2 UU_{x}+\alpha _3 U_{xxx}=0, \end{aligned}$$

that has been thoroughly examined by several authors. By using the transformation $U(x,t)=w^{2}(x,t)$. Then Eq. (1) can be written as

4 $$\begin{aligned} 2ww_t +2\alpha _1 w^{2}w_x +2\alpha _2 w^{3}w_x+ 6\alpha _3 w_x w_{xx}+2\alpha _3 ww_{xxx}=0. \end{aligned}$$

Here we study this equation from the point of view of Lie symmetry analysis. Our main concern is to discuss the optimal system for the S-KdV Eq. (4) and then to utilize it for symmetry reductions to obtain analytical solutions. Our findings will include periodic solutions, double periodic, shock waves, bell-shaped, and solitary waves. Lie symmetry method is a powerful method and is popular among recent techniques. This technique effectively deals with nonlinear PDEs. This method has been used for the study of vast nonlinear problems, especially the study of micromorphic model^19^, Riabouchinsky Proudman Johnson equation^20^, generalized Pochhammer–Chree equation^21^, the Benney–Luke equation^22^, Peyrard–Bishop model^23^ and many others^24, 25^. The literature for the Lie symmetry analysis is well documented and one can see^26–28^.

This article is organized as follows: in “Lie symmetries”, we give the optimal system (one-dimensional) and the Lie symmetry analysis. Utilizing the optimal system of one-dimensional subalgebras, we can produce the analytical solutions in “Invariant solutions and symmetry reductions”. In “Generalized JEF method” and “A description of the MAE procedure”, we examine solitary wave solutions based on trigonometric, hyperbolic, exponential, and rational function solitary solutions. “Physical interpretations of the solutions” addresses physical interpretation, and the conclusion follows.

## Lie symmetries

Let us consider a one-parameter (local) Lie group of transformations ($\vartheta$ parameter) given by

5 $$\begin{aligned} \begin{aligned} \tilde{x}\rightarrow x+\vartheta \varphi _{1}(x,t,w)+O(\vartheta ^{2}),\\ \tilde{t}\rightarrow t+\vartheta \varphi _{2}(x,t,w)+O(\vartheta ^{2}),\\ \tilde{w}\rightarrow w+\vartheta \eta (x,t,w)+O(\vartheta ^{2}). \end{aligned} \end{aligned}$$

The infinitesimal generator associated with the above transformations is

6 $$\begin{aligned} \Sigma =\varphi _{1}(x,t,w)\frac{\partial }{\partial x}+\varphi _{2}(x,t,w)\frac{\partial }{\partial t}+\eta (x,t,w) \frac{\partial }{\partial w}\cdot \end{aligned}$$

The coefficient functions $\varphi _{1}, \varphi _{2}$ and $\eta$ are to be found, and using the Lie invariance condition

7 $$\begin{aligned} \Sigma ^{[3]}(\theta )|_{\theta =0}=0, \end{aligned}$$

where $\Sigma ^{[3]}$ is the third extension of $\Sigma$ and

$$\begin{aligned} \theta =2ww_t +2\alpha _1 w^{2}w_x +2\alpha _2 w^{3}w_x+ 6\alpha _3 w_x w_{xx}+2\alpha _3 ww_{xxx}. \end{aligned}$$

By solving the determining equations originating from Eq. (7), we can derive the symmetry generators of Eq. (4),

**Case-1**  $$(\alpha _1,\alpha _2 \ne 0)$$:

$$\begin{aligned} \Sigma _1 =\frac{\partial }{\partial t},~ \Sigma _2 =\frac{\partial }{\partial x}\cdot \end{aligned}$$

**Case-2**  $$(\alpha _2 = 0)$$:

$$\begin{aligned} \Sigma _1 =\frac{\partial }{\partial t},~ \Sigma _2 =\frac{\partial }{\partial x},~\Sigma _3 =x\frac{\partial }{\partial x}+3t\frac{\partial }{\partial t}-2w\frac{\partial }{\partial w}\cdot \end{aligned}$$

**Case-3**  $$(\alpha _1 =0)$$:

$$\begin{aligned} \Sigma _1 =\frac{\partial }{\partial t},~ \Sigma _2 =\frac{\partial }{\partial x},~\Sigma _3 =2\alpha _2 t\frac{\partial }{\partial x}+\frac{1}{w}\frac{\partial }{\partial w},~\Sigma _4 =x\frac{\partial }{\partial x}+3t\frac{\partial }{\partial t}-w\frac{\partial }{\partial w}\cdot \end{aligned}$$

### One-dimensional optimal systems

The idea of an optimal system of subalgebras for a certain Lie algebra to obtain fundamentally distinct invariant solutions was perhaps originally introduced by Ovsyannikov^27^. Then it was followed by Ibragimov^28^ and continued by Olver^29^. To determine the optimal system, we identify the sets of equivalent classes of one-dimensional subalgebras by observing their behavior under the influence of the adjoint representation.

The representation of the adjoint action can be expressed as,

8 $$\begin{aligned} Ad(\exp {(\vartheta \Sigma _m )}\cdot \Sigma _n) = \Sigma _n -\vartheta [\Sigma _m, \Sigma _n ]+\frac{\vartheta ^{2}}{2!}[\Sigma _m,[\Sigma _m, \Sigma _n ]]-\cdots , \end{aligned}$$

where $\vartheta$ represents a real number and $[\Sigma _m , \Sigma _n ]$ indicates the Lie product defined by,

9 $$\begin{aligned}{}[\Sigma _m, \Sigma _n ]=\Sigma _m \Sigma _n -\Sigma _n \Sigma _m. \end{aligned}$$

#### Optimal system for Case-1

In this case, the algebra is two-dimensional and satisfies the commutation relation,

$$\begin{aligned} {[}\Sigma _1,\Sigma _2 ]=0. \end{aligned}$$

We take into account following general element $\Sigma$ of symmetry algebra $\textrm{L}_{2}$ given by,

10 $$\begin{aligned} \Sigma =\lambda _{1}\Sigma _1 +\lambda _{2}\Sigma _2. \end{aligned}$$

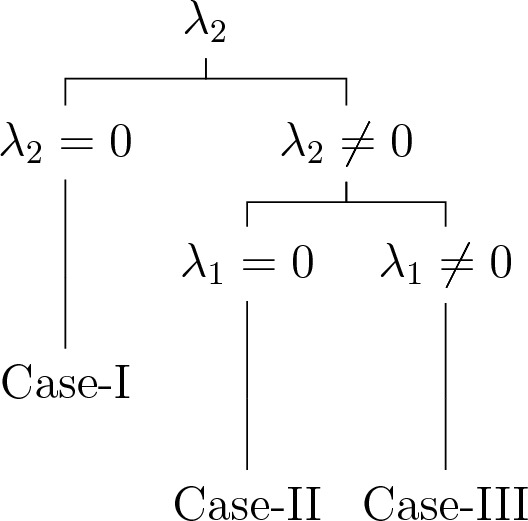


When taking into account that the commutator relations are equal to zero, it becomes clear that the vector form cannot be further simplified.

**Case-I**. $\lambda _{2}= 0$, so that

11 $$\begin{aligned} \Sigma =\lambda _{1}\Sigma _1. \end{aligned}$$

We get

$$\begin{aligned} \mathcal {Q}_{1}=\Sigma _1, \end{aligned}$$

**Case-II**. $\lambda _1 = 0,\lambda _{2}\ne 0$, so that

12 $$\begin{aligned} \Sigma =\lambda _{2}\Sigma _2. \end{aligned}$$

We have

$$\begin{aligned} \mathcal {Q}_{2}=\Sigma _2, \end{aligned}$$

**Case-III**. $\lambda _1 \ne 0,\lambda _{2} \ne 0$, so that

13 $$\begin{aligned} \Sigma =\lambda _{1}\Sigma _1 +\lambda _{2}\Sigma _2. \end{aligned}$$

We get

$$\begin{aligned} \mathcal {Q}_{3}=\Sigma _1 +c \Sigma _2,\quad c \ne 0. \end{aligned}$$

Therefore, the set of symmetry subalgebras representing the optimal system can be described as follows

14 $$\begin{aligned} \begin{aligned} \mathcal {Q}_1&=\Sigma _1,\\ \mathcal {Q}_2&=\Sigma _2,\\ \mathcal {Q}_3&=\Sigma _1 +c\Sigma _2,~~c\ne 0.\\ \end{aligned} \end{aligned}$$

#### Optimal system for Case-2

In this case, the algebra is three-dimensional with non-zero commutators,

$$\begin{aligned} {[}\Sigma _1,\Sigma _3 ] =3\Sigma _1,~[\Sigma _2,\Sigma _3 ]=\Sigma _2. \end{aligned}$$

Table 1 displays the adjoint table, which assists us in calculating the optimal system of one-dimensional subalgebras.

**Table 1:** Adjoint table.

$Ad(e^{\vartheta })$	$\Sigma _{1}$	$\Sigma _{2}$	$\Sigma _{3}$
${\Sigma _1}$	${\Sigma _1}$	${\Sigma _2}$	${\Sigma _3}-3\vartheta \Sigma _1$
${\Sigma _2}$	${\Sigma _1}$	${\Sigma _2}$	${\Sigma _3}-\vartheta \Sigma _2$
${\Sigma _3}$	$e^{3\vartheta }{\Sigma _1}$	$e^{\vartheta }{\Sigma _2}$	${\Sigma _3}$

Consider an arbitrary element $\Sigma$ of symmetry algebra $\textrm{L}_{3}$ given by,

15 $$\begin{aligned} \Sigma =\lambda _{1}\Sigma _1 +\lambda _{2}\Sigma _2 +\lambda _3 \Sigma _3. \end{aligned}$$

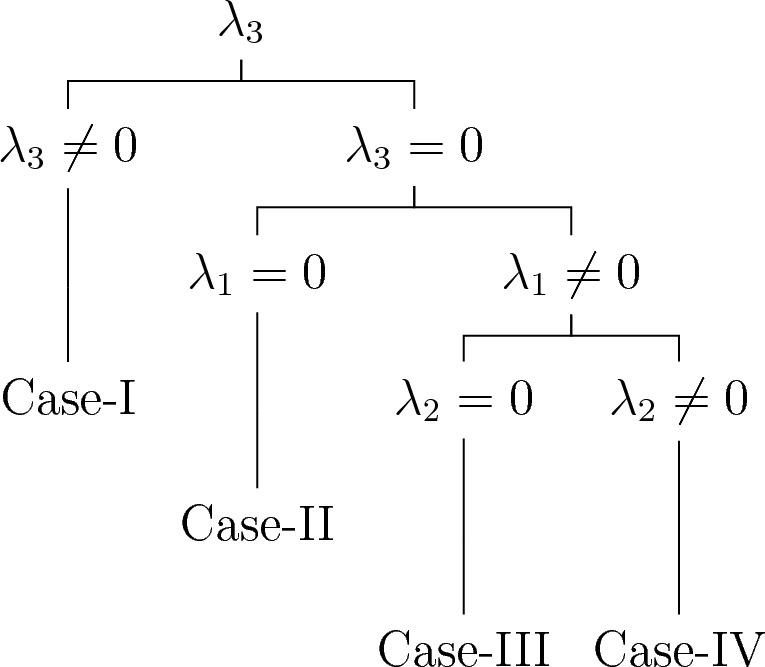


**Case-I**. $\lambda _3 \ne 0$, so that

16 $$\begin{aligned} \Sigma= & {} \lambda _{1}\Sigma _1 +\lambda _{2}\Sigma _2 +\lambda _3 \Sigma _3 \end{aligned}$$

17 $$\begin{aligned} \Sigma '= & {} Ad(e^{\vartheta }\Sigma _1 )\Sigma =\lambda _{2}\Sigma _2 +\lambda _3 \Sigma _3 \end{aligned}$$

18 $$\begin{aligned} \Sigma ''= & {} Ad(e^{\vartheta }\Sigma _1 )\Sigma ' =\lambda _3 \Sigma _3 \end{aligned}$$

We get

$$\begin{aligned} \mathcal {Q}_{1}=\Sigma _3 \end{aligned}$$

**Case-II.** $\lambda _1 = 0,\lambda _3 =0$, so that

19 $$\begin{aligned} \Sigma =\lambda _{2}\Sigma _2 \end{aligned}$$

We get

$$\begin{aligned} \mathcal {Q}_{2}=\Sigma _2 \end{aligned}$$

**Case-III.** $\lambda _1 \ne 0,\lambda _3 =0,\lambda _2 =0$, so that

20 $$\begin{aligned} \Sigma =\lambda _{1}\Sigma _1 \end{aligned}$$

We get

$$\begin{aligned} \mathcal {Q}_{3}=\Sigma _1 \end{aligned}$$

**Case-IV.** $\lambda _1 \ne 0,\lambda _2 \ne 0,\lambda _3 =0$, so that

21 $$\begin{aligned} \Sigma= & {} \lambda _{1}\Sigma _1 +\lambda _2 \Sigma _2 \end{aligned}$$

22 $$\begin{aligned} \Sigma '= & {} Ad(e^{\vartheta }\Sigma _3 )\Sigma =\lambda _{1}\Sigma _1 +e^{-2\vartheta }\lambda _2 \Sigma _2 \end{aligned}$$

We get

$$\begin{aligned} \mathcal {Q}_{4}=\Sigma _1 \pm \Sigma _2 \end{aligned}$$

Therefore, the set of symmetry subalgebras representing the optimal system can be described as follows

23 $$\begin{aligned} \begin{aligned} \mathcal {Q}_1&=\Sigma _3,\\ \mathcal {Q}_2&=\Sigma _2,\\ \mathcal {Q}_3&=\Sigma _1,\\ \mathcal {Q}_4&=\Sigma _1 \pm \Sigma _2. \end{aligned} \end{aligned}$$

#### Optimal system for Case-3

In this case, the algebra is four dimensional with nonzero commutators,

$$\begin{aligned} {[}\Sigma _1,\Sigma _3 ] =2\alpha _2 \Sigma _2,~[\Sigma _1,\Sigma _4 ]=3\Sigma _1,~[\Sigma _2,\Sigma _4 ]=\Sigma _2,~ [\Sigma _3,\Sigma _4 ]=-2\Sigma _3 \end{aligned}$$

Table 2 displays the adjoint table, which assists us in calculating the optimal system of one-dimensional subalgebras.

**Table 2:** Adjoint table.

$Ad(e^{\vartheta })$	$\Sigma _{1}$	$\Sigma _{2}$	$\Sigma _{3}$	$\Sigma _4$
${\Sigma _1}$	${\Sigma _1}$	${\Sigma _2}$	${\Sigma _3}-2\alpha _2 \vartheta \Sigma _2$	$\Sigma _4 -3\vartheta \Sigma _1$
${\Sigma _2}$	${\Sigma _1}$	${\Sigma _2}$	${\Sigma _3}$	$\Sigma _4 -\vartheta \Sigma _2$
${\Sigma _3}$	${\Sigma _1}+2\alpha _2 \vartheta \Sigma _2$	${\Sigma _2}$	${\Sigma _3}$	$\Sigma _4 +2\vartheta \Sigma _3$
${\Sigma _4}$	$e^{3\vartheta }{\Sigma _1}$	$e^{\vartheta }{\Sigma _2}$	$e^{-2\vartheta }{\Sigma _3}$	$\Sigma _4$

Consider an arbitrary element $\Sigma$ of symmetry algebra $\textrm{L}_{4}$ given by,

24 $$\begin{aligned} \Sigma =\lambda _{1}\Sigma _1 +\lambda _{2}\Sigma _2 +\lambda _3 \Sigma _3 +\lambda _4 \Sigma _4. \end{aligned}$$

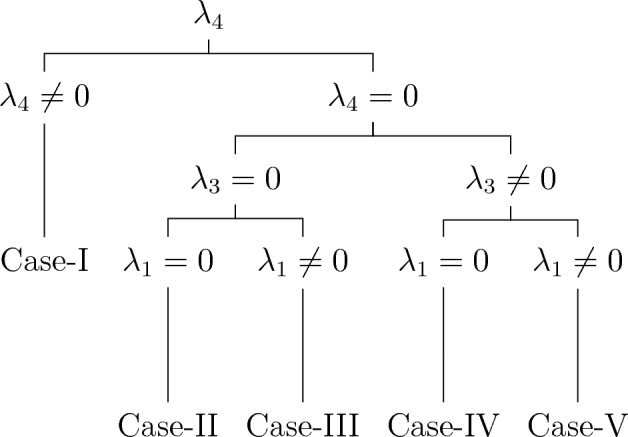


**Case-I.** $\lambda _4 \ne 0$, so that

25 $$\begin{aligned} \Sigma= & {} \lambda _{1}\Sigma _1 +\lambda _{2}\Sigma _2 +\lambda _3 \Sigma _3 +\lambda _4 \Sigma _4 \end{aligned}$$

26 $$\begin{aligned} \Sigma '= & {} Ad(e^{\vartheta }\Sigma _1 )\Sigma =\lambda _{3}\Sigma _3 +\lambda _4 \Sigma _4 \end{aligned}$$

27 $$\begin{aligned} \Sigma ''= & {} Ad(e^{\vartheta }\Sigma _3 )\Sigma ' =\lambda _4 \Sigma _4. \end{aligned}$$

We get

$$\begin{aligned} \mathcal {Q}_{1}=\Sigma _4. \end{aligned}$$

**Case-II.** $\lambda _1 = 0, \lambda _3 =0, \lambda _4 =0$, so that

28 $$\begin{aligned} \Sigma =\lambda _{2}\Sigma _2 \end{aligned}$$

We get

$$\begin{aligned} \mathcal {Q}_{2}=\Sigma _2. \end{aligned}$$

**Case-III.** $\lambda _1 \ne 0, \lambda _3 =0, \lambda _4 =0$, so that

29 $$\begin{aligned} \Sigma= & {} \lambda _{1}\Sigma _1 +\lambda _2 \Sigma _2. \end{aligned}$$

30 $$\begin{aligned} \Sigma '= & {} Ad(e^{\vartheta }\Sigma _3 )\Sigma =\lambda _1 \Sigma _1 \end{aligned}$$

We get

$$\begin{aligned} \mathcal {Q}_{3}=\Sigma _1. \end{aligned}$$

**Case-IV.** $\lambda _1 = 0, \lambda _3 \ne 0, \lambda _4 =0$, so that

31 $$\begin{aligned} \Sigma= & {} \lambda _{2}\Sigma _2 +\lambda _3 \Sigma _3 \end{aligned}$$

32 $$\begin{aligned} \Sigma '= & {} Ad(e^{\vartheta }\Sigma _1 )\Sigma =\lambda _3 \Sigma _3 \end{aligned}$$

We get

$$\begin{aligned} \mathcal {Q}_{4}=\Sigma _3. \end{aligned}$$

**Case-V.** $\lambda _1 \ne 0, \lambda _3 \ne 0, \lambda _4 =0$, so that

33 $$\begin{aligned} \Sigma= & {} \lambda _1 \Sigma _1 +\lambda _{2}\Sigma _2 +\lambda _3 \Sigma _3 \end{aligned}$$

34 $$\begin{aligned} \Sigma '= & {} Ad(e^{\vartheta }\Sigma _3 )\Sigma =\lambda _1 \Sigma _1 +\lambda _3 \Sigma _3 \end{aligned}$$

35 $$\begin{aligned} \Sigma ''= & {} Ad(e^{\vartheta }\Sigma _4 )\Sigma ' =\lambda _1 \Sigma _1 +e^{-2\vartheta }\lambda _3 \Sigma _3 \end{aligned}$$

We get

$$\begin{aligned} \mathcal {Q}_{5}=\Sigma _1 \pm \Sigma _3. \end{aligned}$$

Therefore, the set of symmetry subalgebras representing the optimal system can be described as follows

36 $$\begin{aligned} \begin{aligned} \mathcal {Q}_1&=\Sigma _4,\\ \mathcal {Q}_2&=\Sigma _2,\\ \mathcal {Q}_3&=\Sigma _1,\\ \mathcal {Q}_4&=\Sigma _3,\\ \mathcal {Q}_5&=\Sigma _1 \pm \Sigma _3. \end{aligned} \end{aligned}$$

## Invariant solutions and symmetry reductions

### Symmetry reductions for Case-1

**Case-I.** Consider $\mathcal {Q}_1 =\Sigma _1$. The associated Lagrange equation is

$$\begin{aligned} \frac{dt}{1}=\frac{dx}{0}=\frac{dw}{0}\cdot \end{aligned}$$

We obtain the similarity variables $w=\tau (\theta ), \theta =x$. By using this transformation, it becomes possible to express the simplified version of Eq. (4) in the following manner

37 $$\begin{aligned} \alpha _2 \tau ^{3}\tau '+\alpha _1 \tau ^{2}\tau '+\alpha _3 \tau \tau '''+3\alpha _3 \tau ' \tau ''=0. \end{aligned}$$

We recommend obtaining a numerical solution for the aforementioned ODE.

**Case-II.** Consider $\mathcal {Q}_2 =\Sigma _2$. The associated Lagrange equation is

$$\begin{aligned} \frac{dt}{0}=\frac{dx}{1}=\frac{dw}{0}\cdot \end{aligned}$$

We obtain the similarity variables $w=\tau (\theta ), \theta =t$. By using this transformation, it becomes possible to express the simplified version of Eq. (4) in the following manner

38 $$\begin{aligned} 2\tau \tau '=0. \end{aligned}$$

Since, $\tau \ne 0,$ this imples $\tau '=0$ which gives $\tau (\theta )=c_1.$ Hence, exact solution of (4) invariant under $\Sigma _2$ is

39 $$\begin{aligned} w(x,t)=c_1. \end{aligned}$$

**Case-III.** Consider $\mathcal {Q}_3 =\Sigma _1 +c\Sigma _2$. The associated Lagrange equation is

$$\begin{aligned} \frac{dt}{1}=\frac{dx}{c}=\frac{dw}{0}\cdot \end{aligned}$$

We obtain the similarity variables $w=\tau (\theta ) , \theta =t-\frac{x}{c}$. By using this transformation, it becomes possible to express the simplified version of Eq. (4) in the following manner

40 $$\begin{aligned} -2\alpha _3 \tau \tau '''+2\tau ' (-3\alpha _3 \tau '' +c^{2}\tau (-\alpha _2 \tau ^{2}-\alpha _1 \tau +c))=0. \end{aligned}$$

### Symmetry reductions for Case-2

**Case-I.** Consider $\mathcal {Q}_2 =\Sigma _2$. The associated Lagrange equation is

$$\begin{aligned} \frac{dt}{0}=\frac{dx}{1}=\frac{dw}{0}\cdot \end{aligned}$$

We obtain the similarity variables $w=\tau (\theta ), \theta =t$. By using this transformation, it becomes possible to express the simplified version of Eq. (4) in the following manner

41 $$\begin{aligned} 2\tau \tau '=0. \end{aligned}$$

Since, $\tau \ne 0,$ this imples $\tau '=0$ which gives $\tau (\theta )=c_1.$ Hence, exact solution of Eq. (4) invariant under $\Sigma _2$ is

42 $$\begin{aligned} w(x,t)=c_1. \end{aligned}$$

**Case-II.** Consider $\mathcal {Q}_3 =\Sigma _1$. The associated Lagrange equation is

$$\begin{aligned} \frac{dt}{1}=\frac{dx}{0}=\frac{dw}{0}\cdot \end{aligned}$$

We obtain the similarity variables $w=\tau (\theta ), \theta =x$. By using this transformation, it becomes possible to express the simplified version of Eq. (4) in the following manner

43 $$\begin{aligned} 2\alpha _1 \tau ^{2}\tau '+2\alpha _3 \tau \tau '''+6\alpha _3 \tau ' \tau ''=0. \end{aligned}$$

We recommend obtaining a numerical solution for the aforementioned ODE.

**Case-III.** Consider $\mathcal {Q}_4 =\Sigma _1 +\Sigma _2$. The associated Lagrange equation is

$$\begin{aligned} \frac{dt}{1}=\frac{dx}{1}=\frac{dw}{0}\cdot \end{aligned}$$

We obtain the similarity variables $w=\tau (\theta ), \theta =t-x$. By using this transformation, it becomes possible to express the simplified version of Eq. (4) in the following manner

44 $$\begin{aligned} -2\alpha _3 \tau \tau '''-2\tau '(\alpha _1 \tau ^{2}+3\alpha _3 \tau ''-\tau )=0. \end{aligned}$$

This particular ODE cannot be solved using conventional analytical methods. Therefore, we recommend approaching it through numerical methods.

**Case-IV.** Consider $\mathcal {Q}_4 =\Sigma _1 -\Sigma _2$. The associated Lagrange equation is

$$\begin{aligned} \frac{dt}{1}=\frac{dx}{-1}=\frac{dw}{0}\cdot \end{aligned}$$

We obtain the similarity variables $w=\tau (\theta ), \theta =t+x$. By using this transformation, it becomes possible to express the simplified version of Eq. (4) in the following manner

45 $$\begin{aligned} 2\alpha _3 \tau \tau '''+2\tau '(\alpha _1 \tau ^{2}+3\alpha _3 \tau ''+\tau )=0. \end{aligned}$$

We propose solving this ODE using numerical methods.

### Symmetry reductions for Case-3

**Case-I.**

 Consider $\mathcal {Q}_2 =\Sigma _2$. The associated Lagrange equation is

$$\begin{aligned} \frac{dt}{0}=\frac{dx}{1}=\frac{dw}{0}\cdot \end{aligned}$$

We obtain the similarity variables $w=\tau (\theta ), \theta =t$. By using this transformation, it becomes possible to express the simplified version of Eq. (4) in the following manner

46 $$\begin{aligned} 2\tau \tau '=0. \end{aligned}$$

Since, $\tau \ne 0,$ this imples $\tau '=0$ which gives $\tau (\theta )=c_1.$ Hence, exact solution of (4) invariant under $\Sigma _2$ is

47 $$\begin{aligned} w(x,t)=c_1. \end{aligned}$$

**Case-II.** Consider $\mathcal {Q}_4 =\Sigma _3$. The associated Lagrange equation is

$$\begin{aligned} \frac{dt}{0}=\frac{dx}{2\alpha _2 t}=\frac{dw}{\frac{1}{w}}\cdot \end{aligned}$$

We obtain the similarity variables $w=\frac{\sqrt{\alpha _2 t \tau (\theta )+x}}{\sqrt{\alpha _2}\sqrt{t}} , \theta =t$. By using this transformation, it becomes possible to express the simplified version of Eq. (4) in the following manner

48 $$\begin{aligned} \theta \tau '+\tau =0. \end{aligned}$$

This gives,

49 $$\begin{aligned} \tau (\theta )=\frac{c_1 }{\theta }\cdot \end{aligned}$$

Hence, exact solution of (4) invariant under $\Sigma _3$ is

50 $$\begin{aligned} w(x,t)=\frac{\sqrt{\alpha _2 c_1 +x}}{\sqrt{\alpha _2}\sqrt{t}}\cdot \end{aligned}$$

**Case-III.** Consider $\mathcal {Q}_3 =\Sigma _1$. The associated Lagrange equation is

$$\begin{aligned} \frac{dt}{1}=\frac{dx}{0}=\frac{dw}{0}\cdot \end{aligned}$$

We obtain the similarity variables $w=\tau (\theta ), \theta =x$. By using this transformation, it becomes possible to express the simplified version of Eq. (4) in the following manner

51 $$\begin{aligned} \alpha _2 \tau ^{3}\tau '+\alpha _3 \tau \tau '''+3\alpha _3 \tau ' \tau ''=0. \end{aligned}$$

**Case-IV.** Consider $\mathcal {Q}_5 =\Sigma _1 +\Sigma _3$. The associated Lagrange equation is

$$\begin{aligned} \frac{dt}{1}=\frac{dx}{2\alpha _2 t}=\frac{dw}{\frac{1}{w}}\cdot \end{aligned}$$

We obtain the similarity variables $w=\sqrt{\tau (\theta )+2t} , ~\theta =t^{2}-\frac{x}{\alpha _2}$. By using this transformation, it becomes possible to express the simplified version of Eq. (4) in the following manner

52 $$\begin{aligned} \alpha _3 \tau '''{\alpha _2}^{3}(\tau \tau '-2)=0. \end{aligned}$$

If $\tau '''=0,$ this implies $\tau (\theta )=\frac{c_1}{2}\theta ^{2}+c_2 \theta +c_3.$ So, the solution of (4) in main variables becomes

53 $$\begin{aligned} w(x,t)=\sqrt{\frac{c_1}{2}\Big (t^{2}-\frac{x}{\alpha _2}\Big )^{2}+c_2 \Big (t^{2}-\frac{x}{\alpha _2}\Big ) +c_3 +2t}. \end{aligned}$$

If $\tau '''\ne 0$, then $\tau \tau '-2=0$ this gives $\tau (\theta )=\sqrt{c_1 +4\theta }.$

 Hence, the solution of (4) in main variables becomes

54 $$\begin{aligned} w(x,t)=\sqrt{\sqrt{4\Big (t^{2}-\frac{x}{\alpha _2}\Big )}+c_1}. \end{aligned}$$

**Case-V.** Consider $\mathcal {Q}_5 =\Sigma _1 -\Sigma _3$. The associated Lagrange equation is

$$\begin{aligned} \frac{dt}{1}=\frac{dx}{-2\alpha _2 t}=\frac{dw}{-\frac{1}{w}}\cdot \end{aligned}$$

We obtain the similarity variables $w=\sqrt{\tau (\theta )-2t} , ~\theta =t^{2}+\frac{x}{\alpha _2}$. By using this transformation, it becomes possible to express the simplified version of Eq. (4) in the following manner

55 $$\begin{aligned} \alpha _3 \tau '''+{\alpha _2}^{3}(\tau \tau '-2)=0. \end{aligned}$$

In the next sections, we introduce some methods to deal with reduced ODEs arising in the reductions.

## Generalized JEF method

In this section, we invoke the main steps for the generalized JEF method. This method is very efficient in generating periodic, double periodic, solitary wave, rational, and exponential function solutions. This method has a wide range of applications in engineering, biological sciences, chemical sciences, and mathematical physics. We briefly mention here the algorithm for the generalized JEF method.

**(Step 1):** Consider a nonlinear PDE in the explicit form of the dependent variable

56 $$\begin{aligned} X(w,w_{x},w_{t}, w_{xx},w_{xt},\ldots )=0. \end{aligned}$$

**(Step 2):** We look for the transformation based on scaling and translation given by

57 $$\begin{aligned} w(x,t)=\tau (\theta ),~\theta =x-V t. \end{aligned}$$

In the above equation, the parameter *V* indicates the speed of the wave, and for the stationary solutions $V=0$. Note that Eq. (56) is invariant under the transformation (57) this usually works as an existence criterion for the waveform solutions. The transformation (57) reduces the number of independent variables in the nonlinear PDE (56) and turns it into an ODE of the form

58 $$\begin{aligned} Y(\tau ,\tau ',\tau '',\ldots )=0. \end{aligned}$$

This ODE (58) helps us to classify the waveform solutions of the nonlinear PDE (56).

**(Step 3):** At this stage, we consider the general assumed solution for the ODE (58) as follows

59 $$\begin{aligned} \tau (\theta )=\sum _{q=0}^{N}b_{q}S^q(\theta ). \end{aligned}$$

In the equation provided, the constants $b_q$ (where $q=1, 2, \ldots , N$) represent coefficient values, and the function $S(\theta )$ must fulfill the subsequent Jacobi elliptic equation

60 $$\begin{aligned} S'(\theta )=\sqrt{a_1+a_2S^2(\theta )+\frac{a_3}{2}S^4(\theta )}, \end{aligned}$$

the coefficients $a_1,a_2$ and $a_3$ are nonzero real parameters. The values of these parameters are listed in Table 3.

**(Step 4):** Subsequently, the balance principle is applied to determine the appropriate balancing number *N*.

**(Step 5):** By solving the system described as $S^q=0$ for all values of *q*, we obtain a collection of parameters.

The subsequent relations are valid for the elliptic functions;

61 $$\begin{aligned}{}&{\text {sn}}^{2}(\theta )+{\text {cn}}^{2}(\theta )=1,~{\text {dn}}^{2}(\theta )+\zeta ^{2}{\text {sn}}^{2}(\theta )=1, ~({\text {sn}}(\theta ))'={\text {cn}}(\theta ){\text {dn}}(\theta ), \\&({\text {cn}}(\theta ))'=-{\text {sn}}(\theta ){\text {dn}}(\theta ),~~({\text {dn}}(\theta ))'=-\zeta ^{2}{\text {sn}}(\theta ){\text {cn}}(\theta ). \end{aligned}$$

Here we use ${\text {sn}}(\theta )={\text {sn}}(\theta |\zeta )$.

Trigonometric functions are typical of elliptic functions for $\zeta \mapsto 0$, whereas Table 4 shows examples of hyperbolic functions for $\zeta \mapsto 1$.

**Table 3:** Types of solutions of (60).

$\text {No.}$	$a_1$	$a_2$	$a_{3}$	$S(\theta )$
1	1	$-(1+\zeta ^2)$	$2\zeta ^2$	$\text {sn}(\theta )$
2	$-\zeta ^2(1-\zeta ^2)$	$2\zeta ^2-1$	2	$\text {ds}(\theta )$
3	$1-\zeta ^2$	$2-\zeta ^2$	2	$\text {cs}(\theta )$
4	$1-\zeta ^2$	$2\zeta ^2-1$	$-2\zeta ^2$	$\text {cn}(\theta )$
5	$\zeta ^2-1$	$2-\zeta ^2$	$-2$	$\text {dn}(\theta )$
6	$\frac{1}{4}$	$\frac{(\zeta ^2-2)}{2}$	$\frac{\zeta ^2}{2}$	$\frac{\text {sn}(\theta )}{1\pm \text {dn}(\theta ) }$
7	$\frac{\zeta ^2}{4}$	$\frac{(\zeta ^2-2)}{2}$	$\frac{\zeta ^2}{2}$	$\frac{\text {sn}(\theta )}{1\pm \text {dn}(\theta ) }$
8	$\frac{-(1-\zeta ^2)^2}{4}$	$\frac{(\zeta ^2+1)}{2}$	$\frac{-1}{2}$	$\eta \text {cn}(\theta )\pm \text {dn}(\theta )$
9	$\frac{\zeta ^2-1}{4}$	$\frac{(\zeta ^2+1)}{2}$	$\frac{\zeta ^2-1}{2}$	$\frac{\text {dn}(\theta )}{1\pm \text {sn}(\theta ) }$
10	$\frac{1-\zeta ^2}{4}$	$\frac{1-\zeta ^2}{2}$	$\frac{1-\zeta ^2}{2}$	$\frac{\text {cn}(\theta )}{1\pm \text {sn}(\theta ) }$
11	$\frac{1}{4}$	$\frac{(1-\zeta ^2)^2}{2}$	$\frac{(1-\zeta ^2)^2}{2}$	$\frac{\text {sn}(\theta )}{\text {dn}(\theta ) \pm \text {cn}(\theta ) }$
12	0	0	2	$\frac{D}{\theta }$
13	0	1	0	$De^{\theta }$

**Table 4:** When $$\zeta \mapsto 1$$.

$\text {No.}$	$a_1$	$a_2$	$a_{3}$	$S(\theta )$
1	1	$-2$	2	$\tanh (\theta )$
2	0	1	2	$\text {csch}(\theta )$
3	0	1	2	$\text {csch}(\theta )$
4	0	1	$-2$	$\text {sech}(\theta )$
5	0	1	$-2$	$\text {sech}(\theta )$
6	$\frac{1}{4}$	$\frac{-1}{2}$	$\frac{1}{2}$	$\frac{\tanh (\theta )}{1\pm \text {sech}(\theta ) }$
7	$\frac{1}{4}$	$\frac{-1}{2}$	$\frac{1}{2}$	$\frac{\tanh (\theta )}{1\pm \text {sech}(\theta ) }$
8	0	1	$\frac{-1}{2}$	$\text {sech}(\theta )\pm \text {sech}(\theta )$
9	0	1	0	$\frac{\text {sech}(\theta )}{1\pm \tanh (\theta ) }$
10	0	0	0	$\frac{\text {sech}(\theta )}{1\pm \tanh (\theta ) }$
11	$\frac{1}{4}$	0	0	$\frac{\tanh (\theta )}{\text {sech}(\theta ) \pm \text {sech}(\theta ) }$
12	0	0	2	$\frac{D}{\theta }$
13	0	1	0	$De^{\theta }$

### Periodic solutions of the S-KdV Eq. (4)

By employing the balance principle to the ODE (40), we determine the balancing number to be $N=1$. Subsequently, the following assumed form arises from Eq. (58)

62 $$\begin{aligned} S(\theta )=b_0+b_1 S(\theta ). \end{aligned}$$

Continuing with step 5 of the algorithm mentioned earlier, we obtain

63 $$\begin{aligned} c=c,~a_0=-\frac{21}{13}c,~a_1=a_1,~\alpha _2=\frac{65}{294c}\cdot \end{aligned}$$

Therefore, Eq. (62) transforms into

64 $$\begin{aligned} S(\theta )=-\frac{21}{13}c+b_1 S(\theta ). \end{aligned}$$

Equation (64) results in a diverse range of solutions for the S-KdV Eq. (4), achieved by altering the parameters as outlined in Table 3.

**Subcase: 1.** When $$a_1=1,\,\,a_2=-(1+\zeta ^2),\,\,\,a_3=2\zeta ^2.$$

For this case, we take $S(\theta )= \text {sn}(\theta ,\zeta )$ to obtain periodic wave solutions of the Eq. (40);

65 $$\begin{aligned} S(\theta )=-\frac{21}{13}c+b_1 {\text {sn}}(\theta ,\zeta ). \end{aligned}$$

When $\zeta$ is set to 1, Eq. (65) transforms into the solution corresponding to a shock wave

66 $$\begin{aligned} S(\theta )=-\frac{21}{13}c+b_1\tanh (\theta ). \end{aligned}$$

By applying the transformation (57), the solution for the S-KdV Eq. (4) can be expressed as

67 $$\begin{aligned} w_1(x,t)= -\frac{21}{13}c+b_1\tanh \left( t-\frac{x}{c}\right) . \end{aligned}$$

**Subcase: 2.** When $$a_1=-\zeta ^2(1-\zeta ^2),\,\,a_2=2\zeta ^2-1,\,\,\,a_3=2.$$

For this case, we take $S(\theta )= \text {ds}(\theta ,\zeta )$ to obtain periodic wave solutions of the Eq. (40);

68 $$\begin{aligned} S(\theta )=-\frac{21}{13}c+b_1\text {ds}(\theta ,\zeta ). \end{aligned}$$

Equation (68) degenerates to $\zeta \mapsto 1$ in this case

69 $$\begin{aligned} S(\theta )=-\frac{21}{13}c+b_1\text {csch}(\theta ). \end{aligned}$$

Employing the transformation (57), the solution for the S-KdV Eq. (4) is obtained as

70 $$\begin{aligned} w_2(x,t)= -\frac{21}{13}c+b_1\text {csch}\left( t-\frac{x}{c}\right) . \end{aligned}$$

**Subcase: 3.** When $$a_1=1-\zeta ^2,\,\,a_2=2-\zeta ^2,\,\,\,a_3=2.$$

For this case, we take $S(\theta )= \text {cs}(\theta ,\zeta )$ to obtain periodic wave solutions of the Eq. (40);

71 $$\begin{aligned} S(\theta )=-\frac{21}{13}c+b_1\text {cs}(\theta ,\zeta ). \end{aligned}$$

Equation (71) degenerates to $\zeta \mapsto 1$ in this case

72 $$\begin{aligned} S(\theta )=-\frac{21}{13}c+b_1\coth (\theta ). \end{aligned}$$

Employing the transformation (57), the solution for the S-KdV Eq. (4) is obtained as

73 $$\begin{aligned} w_3(x,t)= -\frac{21}{13}c+b_1\coth \left( t-\frac{x}{c}\right) . \end{aligned}$$

We follow in a similar way for $\zeta \mapsto 1$

74 $$\begin{aligned} w_4(x,t)= -\frac{21}{13}c+b_1\text {csch}\left( t-\frac{x}{c}\right) . \end{aligned}$$

**Subcase: 4.** When $$a_1=1-\zeta ^2,\,\,a_2=2\zeta ^2-1,\,\,\,a_3=-2\zeta ^2.$$

For this case, we take $S(\theta )= \text {cn}(\theta ,\zeta )$ to obtain periodic wave solutions of the Eq. (40);

75 $$\begin{aligned} S(\theta )=-\frac{21}{13}c+b_1\text {cn}(\theta ,\zeta ). \end{aligned}$$

Equation (75) degenerates to $\zeta \mapsto 1$ in this case

76 $$\begin{aligned} S(\theta )=-\frac{21}{13}c+b_1\text {sech}(\theta ). \end{aligned}$$

Employing the transformation (57), the solution for the S-KdV Eq. (4) is obtained as

77 $$\begin{aligned} w_5(x,t)= -\frac{21}{13}c+b_1\text {sech}\left( t-\frac{x}{c}\right) . \end{aligned}$$

**Subcase: 5.** When $$a_1=\zeta ^2-1,\,\,a_2=2-\zeta ^2,\,\,\,a_3=-2.$$

For this case, we take $S(\theta )= \text {dn}(\theta ,\zeta )$ in order to obtain periodic wave solutions of the Eq. (40);

78 $$\begin{aligned} S(\theta )=-\frac{21}{13}c+b_1\text {dn}(\theta ,\zeta ). \end{aligned}$$

Equation (78) degenerates to $\zeta \mapsto 1$ in this case

79 $$\begin{aligned} S(\theta )=-\frac{21}{13}c+b_1\text {sech}(\theta ). \end{aligned}$$

Employing the transformation (57), the solution for the S-KdV Eq. (4) is obtained as

80 $$\begin{aligned} w_6(x,t)= -\frac{21}{13}c+b_1\text {sech}\left( t-\frac{x}{c}\right) . \end{aligned}$$

**Subcase: 6.** When $$a_1=\frac{1}{4},\,\,a_2=\frac{\zeta ^2-2}{2},\,\,\,a_3=\frac{\zeta ^2}{2}\cdot$$

For this case, we take $S(\theta )= \frac{\text {sn}(\theta ,\zeta )}{1\pm \text {dn}(\theta ,\zeta )}$ in order to obtain periodic wave solutions of the Eq. (40);

81 $$\begin{aligned} S(\theta )=-\frac{21}{13}c+b_1\frac{\text {sn}(\theta ,\zeta )}{1\pm \text {dn}(\theta ,\zeta )}\cdot \end{aligned}$$

Equation (81) degenerates to $\zeta \mapsto 1$ in this case

82 $$\begin{aligned} S(\theta )=-\frac{21}{13}c+b_1\frac{\tanh (\theta )}{1\pm \text {sech}(\theta )}\cdot \end{aligned}$$

Employing the transformation (57), the solution for the S-KdV Eq. (4) is obtained as

83 $$\begin{aligned} w_7(x,t)= -\frac{21}{13}c+b_1\frac{\tanh \left( t-\frac{x}{c}\right) }{1\pm \text {sech}\left( t-\frac{x}{c}\right) }\cdot \end{aligned}$$

**Subcase: 7.** When $$a_1=\frac{\zeta ^2}{4},\,\,a_2=\frac{\zeta ^2-2}{2},\,\,\,a_3=\frac{\zeta ^2}{2}\cdot$$

For this case, we take $S(\theta )= \frac{\text {sn}(\theta ,\zeta )}{1\pm \text {dn}(\theta ,\zeta )}$ to obtain periodic wave solutions of the Eq. (40);

84 $$\begin{aligned} S(\theta )=-\frac{21}{13}c+b_1\frac{\text {sn}(\theta ,\zeta )}{1\pm \text {dn}(\theta ,\zeta )}\cdot \end{aligned}$$

Equation (84) degenerates to $\zeta \mapsto 1$ in this case

85 $$\begin{aligned} S(\theta )=-\frac{21}{13}c+b_1\frac{\tanh (\theta )}{1\pm \text {sech}(\theta )}\cdot \end{aligned}$$

Employing the transformation (57), the solution for the S-KdV Eq. (4) is obtained as

86 $$\begin{aligned} w_8(x,t)=-\frac{21}{13}c+b_1\frac{\tanh \left( t-\frac{x}{c}\right) }{1\pm \text {sech}(t-\frac{x}{c})}\cdot \end{aligned}$$

**Subcase: 8.** When $$a_1=-\frac{(1-\zeta ^2)^2}{4},\,\,a_2=\frac{(\zeta ^2+1)}{2},\,\,\,a_3=\frac{-1}{2}\cdot$$

For this case, we take $S(\theta )= \zeta \text {cn}(\theta ,\zeta )\pm \text {dn}(\theta ,\zeta )$ to obtain periodic wave solutions of the Eq. (40);

87 $$\begin{aligned} S(\theta )=-\frac{21}{13}c+b_1(\zeta \text {cn}(\theta ,\zeta )\pm \text {dn}(\theta ,\zeta )). \end{aligned}$$

Equation (87) degenerates to $\zeta \mapsto 1$ in this case

88 $$\begin{aligned} S(\theta )=-\frac{21}{13}c+b_1(\text {sech}(\theta )\pm \text {sech}(\theta ))\cdot \end{aligned}$$

Employing the transformation (57), the solution for the S-KdV Eq. (4) is obtained as

89 $$\begin{aligned} w_9(x,t)= -\frac{21}{13}c+b_1\left( \text {sech}\left( t-\frac{x}{c}\right) \pm \text {sech}\left( t-\frac{x}{c}\right) \right) . \end{aligned}$$

**Subcase: 9.** When $$a_1=\frac{\zeta ^2-1}{4},\,\,a_2=\frac{\zeta ^2+1}{2},\,\,\,a_3=\frac{\zeta ^2-1}{2}\cdot$$

For this case, we take $S(\theta )= \frac{\text {dn}(\theta ,\zeta )}{1\pm \text {sn}(\theta ,\zeta )}$ to obtain double periodic wave solutions of the Eq. (40);

90 $$\begin{aligned} S(\theta )=-\frac{21}{13}c+b_1\frac{\text {dn}(\theta ,\zeta )}{1\pm \text {sn}(\theta ,\zeta )}\cdot \end{aligned}$$

Equation (90) degenerates to $\zeta \mapsto 1$ in this case

91 $$\begin{aligned} S(\theta )=-\frac{21}{13}c+b_1\frac{\text {sech}(\theta )}{1\pm \tanh (\theta )}\cdot \end{aligned}$$

Employing the transformation (57), the solution for the S-KdV Eq. (4) is obtained as

92 $$\begin{aligned} w_{10}(x,t)= -\frac{21}{13}c+b_1\frac{\text {sech}\left( t-\frac{x}{c}\right) }{1\pm \tanh \left( t-\frac{x}{c}\right) }\cdot \end{aligned}$$

**Subcase: 10.** When $$a_1=\frac{1-\zeta ^2}{4},\,\,a_2=\frac{1-\zeta ^2}{2},\,\,\,a_3=\frac{1-\zeta ^2}{2}\cdot$$

For this case, we take $S(\theta )= \frac{\text {cn}(\theta ,\zeta )}{1\pm \text {sn}(\theta ,\zeta )}$ to obtain double periodic wave solutions of the Eq. (40);

93 $$\begin{aligned} S(\theta )=-\frac{21}{13}c+b_1\frac{\text {cn}(\theta ,\zeta )}{1\pm \text {sn}(\theta ,\zeta )}\cdot \end{aligned}$$

Equation (94) degenerates to $\zeta \mapsto 1$ in this case

94 $$\begin{aligned} S(\theta )=-\frac{21}{13}c+b_1\frac{\text {sech}(\theta )}{1\pm \tanh (\theta )}\cdot \end{aligned}$$

Employing the transformation (57), the solution for the S-KdV Eq. (4) is obtained as

95 $$\begin{aligned} w_{11}(x,t)= -\frac{21}{13}c+b_1\frac{\text {sech}(t-\frac{x}{c})}{1\pm \tanh \left( t-\frac{x}{c}\right) }\cdot \end{aligned}$$

**Case: 11.** When $$a_1=\frac{1}{4},\,\,a_2=\frac{(1-\zeta ^2)^2}{2},\,\,\,a_3=\frac{(1-\zeta ^2)^2}{2}\cdot$$

For this case, we take $S(\theta )= \frac{\text {sn}(\theta ,\zeta )}{\text {dn}(\theta ,\zeta )\pm \text {cn}(\theta ,\zeta )}$ to obtain double periodic wave solutions of the Eq. (40);

96 $$\begin{aligned} S(\theta )=-\frac{21}{13}c+b_1 \frac{\text {sn}(\theta ,\zeta )}{\text {dn}(\theta ,\zeta )\pm \text {cn}(\theta ,\zeta )}\cdot \end{aligned}$$

Equation (96) degenerates to $\zeta \mapsto 1$ in this case

97 $$\begin{aligned} S(\theta )=-\frac{21}{13}c+b_1\frac{\tanh (\theta )}{\text {sech}(\theta )\pm \text {sech}(\theta )}\cdot \end{aligned}$$

Employing the transformation (57), the solution for the S-KdV Eq. (4) is obtained as

98 $$\begin{aligned} w_{12}(x,t)= -\frac{21}{13}c+b_1\frac{\tanh \left( t-\frac{x}{c}\right) }{\text {sech}\left( t-\frac{x}{c}\right) \pm \text {sech}\left( t-\frac{x}{c}\right) }\cdot \end{aligned}$$

**Subcase: 12.** When $$a_1=0,\,\,a_2=0,\,\,\,a_3=2.$$

For this case, we take $S(\theta )= \frac{D}{\theta }$ in order to obtain rational solutions of the Eq. (40);

99 $$\begin{aligned} S(\theta )=-\frac{21}{13}c+b_1\frac{D}{\theta }\cdot \end{aligned}$$

Employing the transformation (57), the solution for the S-KdV Eq. (4) is obtained as

100 $$\begin{aligned} w_{13}(x,t)= -\frac{21}{13}c+b_1\frac{F}{\left( t-\frac{x}{c}\right) }\cdot \end{aligned}$$

**Case: 13.** When $$a_1=0,\,\,a_2=1,\,\,\,a_3=0.$$

For this case, we take $S(\theta )= De^{\theta }$ to obtain exponential functions-based solutions of the Eq. (40);

101 $$\begin{aligned} S(\theta )=-\frac{21}{13}c+b_1 Fe^{\theta }\cdot \end{aligned}$$

Employing the transformation (57), the solution for the S-KdV Eq. (4) is obtained as

102 $$\begin{aligned} w_{14}(x,t)= -\frac{21}{13}c+b_1 Fe^{\left( t-\frac{x}{c}\right) }, \end{aligned}$$

where, in the aforementioned subcases, *F* is a constant.

## A description of the MAE procedure

The algorithm for the MAE procedure^22^ is described here to be used for the soliton solutions for the reduced ODEs. We follow the first two steps of the algorithm for the MAE procedure as we did for the generalized JEF method in the previous section.

**(Step 3):** At this stage, we proceed with the subsequently assumed solution for the ODE (58),

103 $$\begin{aligned} \tau (\theta )=a_0+\sum _{\phi =1}^{N}\left[ a_\phi \left( \Pi ^\tau \right) ^\phi +b_\phi \left( \Pi ^{\tau }\right) ^{-\phi }\right] , \end{aligned}$$

where $\Pi$, $a_\phi$, $b_\phi$, and $a_0$ are arbitrary constants, and $\tau (\theta )$ satisfies the auxiliary equation

104 $$\begin{aligned} \tau ^{\prime }(\theta )=\frac{\Phi _{1}+ \Phi _{2} \Pi ^{-\tau }+ \Phi _{3} \Pi ^\tau }{\ln ( \Pi )}\cdot \end{aligned}$$

In the above equation $\Phi _1$, $\Phi _2$, and $\Phi _3$ are arbitrary constants and $\Pi >0, \Pi \ne 1$. The subsequent solution of the auxiliary Eq. (104) is presented, taking into consideration different cases.

**(Case i)** If $4 \Phi _{1} \Phi _{3}-\Phi _{3}^2<0, ~ \Phi _{3} \ne 0$, then

$$\begin{aligned} \Pi =\frac{-\Phi _{2} +\sqrt{4 \Phi _{1} \Phi _{3}-\Phi _{3}^2 } \tan \left( \frac{\sqrt{4 \Phi _{1} \Phi _{3}-\Phi _{3}^2 } \xi }{2}\right) }{2 \Phi _{3} } \quad \text{ or } \Pi =-\frac{\Phi _{2}+\sqrt{4 \Phi _{1} \Phi _{3}-\Phi _{3}^2} \cot \left( \frac{\sqrt{4 \Phi _{1} \Phi _{3}-\Phi _{3}^2 }\xi }{2}\right) }{2 \Phi _{3} } \end{aligned}$$

**(Case ii)** If $\Phi _{2}^{2}-4 \Phi _{1} \Phi _{3} >0, ~ \Phi _{3} \ne 0$, then

$$\begin{aligned} \Pi =\frac{-\Phi _{2} +\sqrt{\Phi _{2}^{2}-4 \Phi _{1} \Phi _{3} } \tanh \left( \frac{\sqrt{\Phi _{2}^{2}-4 \Phi _{1} \Phi _{3} } \xi }{2}\right) }{2 \Phi _{3} } \quad \text{ or } \Pi =-\frac{\Phi _{2}+\sqrt{\Phi _{2}^{2}-4 \Phi _{1} \Phi _{3}} \coth \left( \frac{\sqrt{\Phi _{2}^{2}-4 \Phi _{1} \Phi _{3} } \xi }{2}\right) }{2 \Phi _{3} } \end{aligned}$$

**(Case iii)** If $\Phi _{2}^{2}-4 \Phi _{1} \Phi _{3} =0$, and $\Phi _{3} \ne 0$, then

$$\begin{aligned} \Pi =-\frac{2+\delta \xi }{2\Phi _{3} \xi }\cdot \end{aligned}$$

**(Step 4):** We then utilize the balance principle to determine the balancing number, denoted as *N*.

**(Step 5):** We proceed by substituting the obtained values into Eq. (56) for the solutions and subsequently, we replace the transformation defined in **step 2**.

Now, we apply the method described above from the MAE procedure to the S-KdV Eq. (4).

### Exact solutions for the S-KdV Eq. (4)

By applying the balance principle to the ordinary differential equation (51), we deduce the balancing number to be $N=1$. Subsequently, the following assumed form emerges from Eq. (103),

105 $$\begin{aligned} \tau (\theta )=a_0+a_1 \Pi ^{\tau (\theta )}+b_1\Pi ^{-\tau (\theta )}. \end{aligned}$$

By substituting the aforementioned assumption into Eq. (51) and utilizing Eq. (104), we can then solve the system $\Pi ^\tau =0$ to obtain the subsequent set of parameters

#### Group (1)

$$\begin{aligned} \beta =-\frac{12\gamma \Phi ^2_1}{b^2_1},~a_0=a_0,~~a_1=0,~~b_1=b_1. \end{aligned}$$

Using the aforementioned set of values, the following solutions for the S-KdV Eq. (4) are obtained;

#### Family (1): Trigonometric function solutions

 If we have $\Xi =\Phi _2^{2}-4 \Phi _1 \Phi _3 <0$, along with the condition $\Phi _3 \ne 0$, the solutions for the S-KdV Eq. (4) follows as

106 $$\begin{aligned} w(x,t)=a_0+b_1\bigg \{ \frac{-\Phi _2+\sqrt{-\Xi }}{2 \Phi _3} \tan \bigg ( \frac{\sqrt{-\Xi } x}{2}\bigg ) \bigg \}^{-1}, \end{aligned}$$

or

107 $$\begin{aligned} w(x,t)=a_0+b_1\bigg \{ \frac{-\Phi _2+\sqrt{-\Xi }}{2 \Phi _3} \cot \bigg ( \frac{\sqrt{-\Xi } x}{2}\bigg ) \bigg \}^{-1}. \end{aligned}$$

#### Family (2): Hyperbolic function solutions

If we have $\Xi =\Phi _2^{2}-4 \Phi _1 \Phi _3 >0$ along with $\Phi _3 \ne 0$, the solution for the S-KdV Eq. (4) follows as

108 $$\begin{aligned} w(x,t)=a_0+b_1\bigg \{ \frac{-\Phi _2+\sqrt{-\Xi }}{2 \Phi _3} \tanh \bigg ( \frac{\sqrt{-\Xi } x}{2}\bigg ) \bigg \}^{-1}, \end{aligned}$$

or

109 $$\begin{aligned} w(x,t)=a_0+b_1\bigg \{ \frac{-\Phi _2+\sqrt{-\Xi }}{2 \Phi _3} \coth \bigg ( \frac{\sqrt{-\Xi } x}{2}\bigg ) \bigg \}^{-1}. \end{aligned}$$

#### Family (3): Rational function solutions

 If we follow the case when $\Xi = \Phi _2^{2}-4 \Phi _1 \Phi _3 =0$ along with $\Phi _3 \ne 0$, the solution for the S-KdV Eq. (4) follows as

110 $$\begin{aligned} w(x,t)=a_0+b_1\bigg \{-\frac{2+\delta x}{2\Phi _3 x} \bigg \}^{-1}\cdot \end{aligned}$$

Following a similar approach, we proceed with the second set of parameters **Group (2)**.

$$\begin{aligned} \beta =-\frac{12\gamma \Phi ^2_3}{a^2_1},~a_0=a_0,~~a_1=a_1,~~b_1=0. \end{aligned}$$

Using the provided set of values, the subsequent solutions for the S-KdV Eq. (4) are obtained;

#### Family (4): Trigonometric function solutions

 If we have $\Xi =\Phi _2^{2}-4 \Phi _1 \Phi _3 <0$, along with the condition $\Phi _3 \ne 0$, the solutions for the S-KdV Eq. (4) follows as

111 $$\begin{aligned} w(x,t)=a_0+a_1\bigg \{ \frac{-\Phi _2+\sqrt{-\Xi }}{2 \Phi _3} \tan \bigg ( \frac{\sqrt{-\Xi } x}{2}\bigg ) \bigg \}, \end{aligned}$$

or

112 $$\begin{aligned} w(x,t)=a_0+a_1\bigg \{ \frac{-\Phi _2+\sqrt{-\Xi }}{2 \Phi _3} \cot \bigg ( \frac{\sqrt{-\Xi } x}{2}\bigg ) \bigg \}^{-1}. \end{aligned}$$

#### Family (5): Hyperbolic function solutions

 If we have $\Xi =\Phi _2^{2}-4 \Phi _1 \Phi _3 >0$ along with $\Phi _3 \ne 0$, the solution for the the S-KdV Eq. (4) follows as

113 $$\begin{aligned} w(x,t)=a_0+a_1\bigg \{ \frac{-\Phi _2+\sqrt{-\Xi }}{2 \Phi _3} \tanh \bigg ( \frac{\sqrt{-\Xi } x}{2}\bigg ) \bigg \}, \end{aligned}$$

or

114 $$\begin{aligned} w(x,t)=a_0+a_1\bigg \{ \frac{-\Phi _2+\sqrt{-\Xi }}{2 \Phi _3} \coth \bigg ( \frac{\sqrt{-\Xi } x}{2}\bigg ) \bigg \}. \end{aligned}$$

#### Family (6): Rational function solutions

If we follow the case when $\Xi = \Phi _2^{2}-4 \Phi _1 \Phi _3 =0$ along with $\Phi _3 \ne 0$, the solution for the S-KdV Eq. (4) follows as

115 $$\begin{aligned} w(x,t)=a_0-a_1\frac{2+\delta x}{2\Phi _3 x} \cdot \end{aligned}$$

## Physical interpretations of the solutions

**Figure 1:**
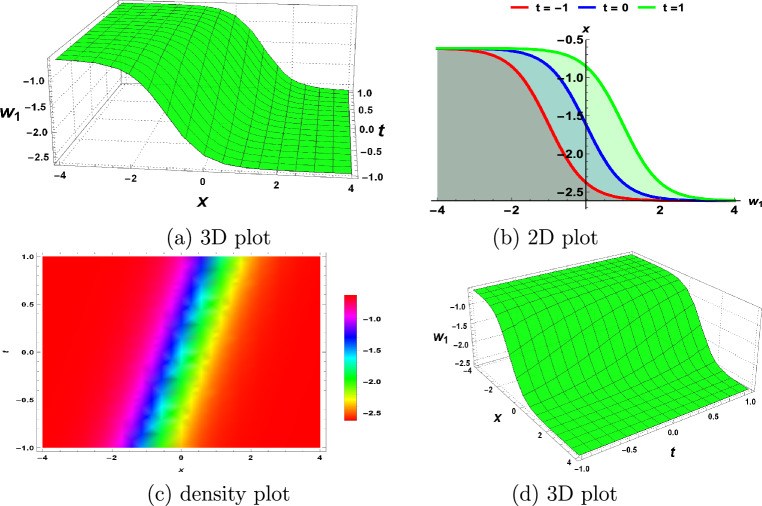
(**a**,**d**) Shock wave nature of the S-KdV Eq (4) via $w_{1}(x,t)$ and the choices of parameters are $c=1,~b_1=1$. (**b**) By taking $w_{1}(x,t)$ at $t=-1,0,1$. (**c**) Density behavior of $w_{1}(x,t)$ in the mentioned region.

**Figure 2:**
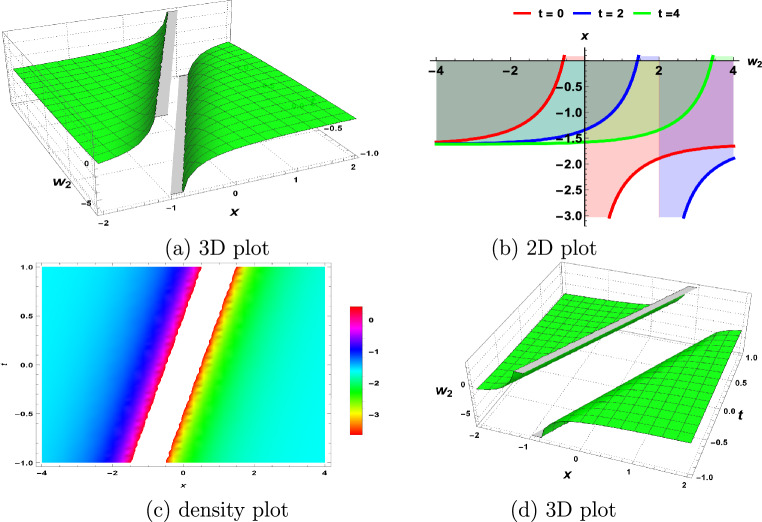
(**a**,**d**) Singular soliton nature of the S-KdV Eq. (4) via $w_{2}(x,t)$ and the choices of parameters are $c=1,~b_1=1$. (**b**) By taking $w_{2}(x,t)$ at $t=0,2,4$. (**c**) Density behavior of $w_{2}(x,t)$ in the mentioned region.

**Figure 3:**
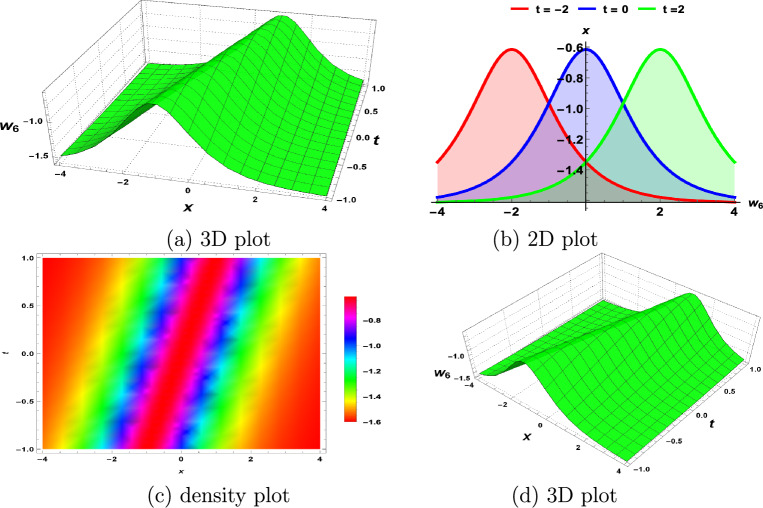
(**a**, **d**) Bell shape nature of the S-KdV Eq (4) via $w_{6}(x,t)$ and the choices of parameters are $c=1,~b_1=1$. (**b**) By taking $w_{6}(x,t)$ at $t=-2,0,2$. (**c**) Density behavior of $w_{6}(x,t)$ in the mentioned region.

**Figure 4:**
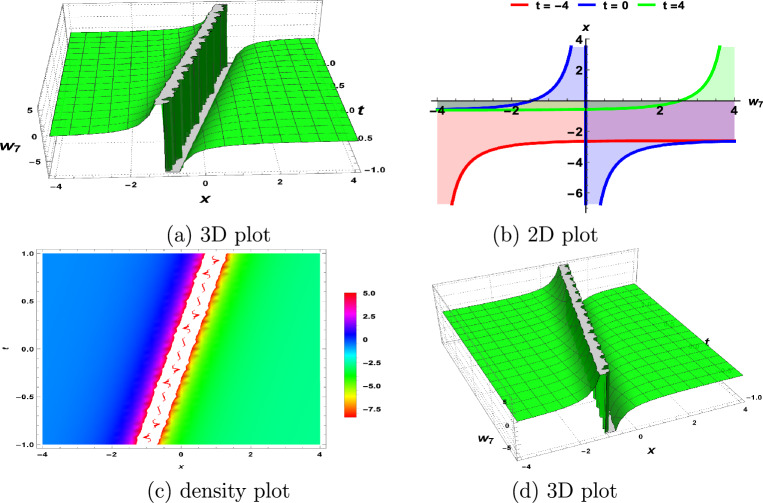
(**a**,**d**) Periodic nature of the S-KdV Eq (4) via $w_{7}(x,t)$ and the choices of parameters are $c=1,~b_1=1$. (**b**) By taking $w_{7}(x,t)$ at $t=-4,0,4$. (**c**) Density behavior of $w_{7}(x,t)$ in the mentioned region.

**Figure 5:**
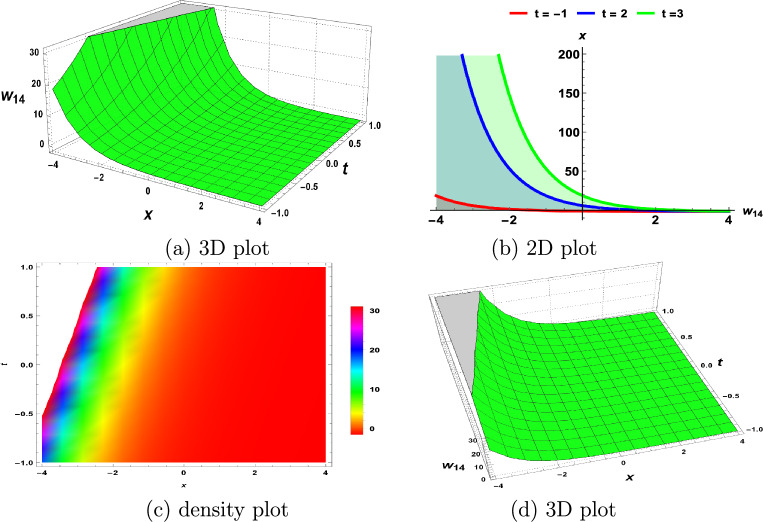
(**a**,**d**) Exponential function nature of the S-KdV Eq. (4) via $w_{14}(x,t)$ and the choices of parameters are $F=1,~c=1,~b_1=1$. (**b**) By taking $w_{14}(x,t)$ at $t=-1,2,3$. (**c**) Density behavior of $w_{14}(x,t)$ in the mentioned region.

In this section, we demonstrate the physical interpretations of the solutions obtained in the above sections. Our findings include rational, trigonometric, hyperbolic and exponential functions-based analytical solutions. The elliptic sine amplitude function solutions lead to the shock wave of the S-KdV Eq. (4) shown in Fig. 1. Similarly, singular soliton nature, bell shape, periodic solutions and exponential natures are shown by Figs. 2, 3, 4, 5 respectively. The Mathematica simulations follow with a certain set of parameters.

## Discussion and conclusion

In this paper, we investigated Lie symmetries and the analytical solutions of the S-KdV equation. The generalized JEF method and the MAE procedure were used to acquire the exact solutions. The solutions include trigonometric, hyperbolic, rational, and exponential functions. The properties of the obtained solutions match with some known structures like periodic wave solutions, bell-shaped solitons or solitary wave solutions, kink-shaped solitons or shock wave solutions and singular soliton solutions. A kink-shaped soliton solution is characterized by boundary values of 0 and $2\pi$ at the left infinity and right infinity, respectively. Our findings prove the soundness of the integrability of the S-KdV Eq. (4) as well as the MAE procedure and the generalized JEF method. The obtained solutions are novel and not reported in the literature in the past.

## Data Availability

All data generated or analyzed during this study are included in this published article [and its supplementary information files].
